# Efficacy and safety of endothelin receptor antagonists, phosphodiesterase type 5 Inhibitors, and prostaglandins in pediatric pulmonary arterial hypertension: A network meta-analysis

**DOI:** 10.3389/fcvm.2022.1055897

**Published:** 2023-01-11

**Authors:** Fen Cao, Kun Wu, Yong-zhi Zhu, Jun-jun Jiang, Gui Zhang, Jun Liu, Ping Xiao, Yang Tian, Wei Zhang, Sheng Zhang, Feng Hou, Zhong-wu Bao

**Affiliations:** ^1^Department of Cardiology, Huaihua First People's Hospital, Huaihua, China; ^2^Department of Neurology, Huaihua First People's Hospital, Huaihua, China

**Keywords:** pediatric pulmonary arterial hypertension, network meta-analysis, endothelin receptor antagonists, phosphodiesterase type 5 Inhibitors, prostaglandins

## Abstract

**Background:**

Pulmonary arterial hypertension (PAH) is a fatal disease characterized by pulmonary vascular remodeling and increased pulmonary artery pressure, leading to impaired lung oxygenation, right heart failure, and even death. Although great advances have been made in PAH-targeted medications for pediatric patients, the efficacy and safety of these treatments are controversial.

**Methods:**

We retrieved relevant articles from electronic databases including PubMed, EMBASE, Web of Science, and Cochrane Library until 12 April 2022. To compare the effectiveness and safety of endothelin receptor antagonists (ERAs), phosphodiesterase type 5 Inhibitors (PDE-5i), and prostaglandins (ProA) in the treatment of pediatric PAH, we investigated six hemodynamic parameters, four respiratory parameters, intensive care unit (ICU) stay duration, length of hospital stay, and two safety outcomes.

**Results:**

A total of 27 randomized controlled trials (RCTs) were included in the meta-analysis with 1,574 pediatric participants. The duration of mechanical ventilation was shorter for patients using bosentan, sildenafil, and ProsA, compared with that for patients using the placebo. Bosentan helped to shorten more time for mechanical ventilation than ProsA did, while ProsA was more effective than sildenafil in this respect. As for the length of stay in the ICU, patients administered by ProsA or sildenafil needed shorter ICU stay, compared to those using the placebo, while ProsA was more effective for shortening ICU stay time. In light of safety outcomes, there was a statistically significant difference between the sildenafil and the placebo group. Sildenafil surpassed ProsA in reducing the incidence of pulmonary hypertension (PH) crisis.

**Conclusions:**

ERAs were more effective than ProsA in shortening the duration of mechanical ventilation, while ProsA were better for shortening the duration of mechanical ventilation and ICU stay than PDE-5i. PDE-5i were found to generate more benefits in decreasing the occurrence of PH crisis, though further investigation is warranted.

**Systematic review registration:**

https://www.crd.york.ac.uk/PROSPERO/display_record.php?RecordID=351505.

## 1. Introduction

Pulmonary arterial hypertension (PAH) is characterized by increased pulmonary artery pressure and pulmonary vascular resistance (PVR). As a severe progressive life-threatening disorder with poor prognosis, PAH is associated with changes in vascular structure and function of pulmonary arteries, triggering impaired lung oxygenation, right heart failure, and even death. Pulmonary hypertension may occur in people of any age, including infants and children. PAH in children shares common features with that in adults (1). However, there are also some differences between them (1–3). Pediatric was updated to include any children with a mean pulmonary artery pressure (mPAP) >20 mmHg at rest by heart catheterization, pulmonary capillary wedge pressure (PCWP) ≤ 15 mmHg as well as indexed pulmonary vascular resistance (PVRi) >3 woods units·m^2^ (4– 6). Sixty-four children per million were diagnosed with PHA annually, and persistent pulmonary hypertension (PPHN) had the highest incidence among newborns (7).

At current, most treatment options for pediatric PAH are based on adult research and clinical data (5, 8, 9). Evidence has shown that the prognosis of children with PAH has improved significantly due to the application of PAH-targeted drugs (6). The present study investigated three types of pulmonary vasodilators for treating pediatric PAH: ERAs (bosentan), PDE5i (sildenafil), and ProsA (treprostinil, iloprost, beraprost sodium, or epoprostenol). Bosentan is a non-selective ERA that can activate endothelin receptor type A and type B to dilate pulmonary blood vessels, therefore improving hemodynamics in patients with PAH (10). Sildenafil is a highly selective PDE-5i that can inhibit PDE-5 and promote endogenous nitric oxide's function to enhance cyclic guanosine monophosphate concentration in pulmonary vascular smooth muscle cells (PASMCs) and alleviate pulmonary vascular remodeling by inhibiting the proliferation of PASMCs (11, 12). ProsA can dilate pulmonary blood vessels and suppress proliferation to improve clinical symptoms in patients with PAH (13). These PHA-targeted medications are likely to improve hemodynamic and respiratory parameters to relieve PAH symptoms (14).

Several trials have confirmed the efficacy and safety of bosentan in children and the Food and Drug Administration (FDA) and the European Medicines Agency (EMA) have approved the use of bosentan for treating pediatric patients with PAH (15–20). Sildenafil was approved by EMA in 2011 for its use in children. However, a warning was issued against sildenafil of a high dose that can increase the risk of mortality, whereas a low-dose treatment appeared to be ineffective (21). In 2014, the FDA announced that sildenafil should not be prescribed for children with PAH, but under some circumstances, the risk profile may justify the appropriate use of sildenafil in individual cases (22). According to the American Thoracic Society and American Heart Association guidelines, sildenafil is recommended for the management of pediatric PAH (23). Recently, a retrospective study found that sildenafil was well tolerated with manageable side effects among children with PHA (24). Treprostinil was approved by FDA for pediatric patients who do not respond to other drugs (25). Some studies reported a great tolerance of treprostinil and epoprostenol with acceptable side effects among many pediatric patients of all ages (26–30).

To sum up, no consensus has been reached on the efficacy and safety of multiple PAH-targeted drugs for pediatric PAH, although a host of studies have investigated pharmacotherapy for children with PAH. Additionally, no network meta-analyses pooling head-to-head evidence have been conducted to compare the therapeutic effects and safety of three types of PAH-targeted agents (ERAs, PDE-5i, and ProsA) in children with PAH. Therefore, we performed a network meta-analysis to generally evaluate the efficacy and safety of these medications in children with PAH. Our meta-analysis will fill the gap in research field by providing sufficient evidence for clinicians to make the optimal choice for each child patient with PAH.

## 2. Methods

The network meta-analysis protocol has been registered with the International Prospective Register of Systematic Reviews Database (PROSPERO). The registration number is CRD42022351505.

### 2.1. Search strategy

The electronic databases we searched included PubMed, Cochrane Library, EMBASE, and Web of Science. All the English publications until 12 April 2022 were selected without any restrictions on the country. We utilized the following subjective terms with corresponding keywords: (“pulmonary arterial hypertension”) AND (“Child” OR “Child, Preschool” OR “Adolescent” OR “Infant”) AND (“Endothelin Receptor Antagonists” OR “Phosphodiesterase 5 Inhibitors” OR “Prostaglandins”). Then, two investigators (FC and KW) screened articles independently by reviewing the title, abstract, and full text. If there were different opinions, we resolved them together through discussion and sought help from the third investigator (YZZ) if necessary.

### 2.2. Inclusion and exclusion criteria

Studies will be included if they met the following inclusion criteria: (1) written in English; (2) subjects were infants, children, and adolescents (postnatal to 18 years); (3) participants were diagnosed as PAH according to ultrasonic cardiogram or right heart catheter confirmation; (4) PAH-targeted medications were compared with placebo or no drug use or traditional therapy. The exclusion criteria were: (1) animal experiments, systematic reviews, meta-analysis, conference reports, letters, guidelines, case reports, insufficient data, and duplicated studies; (2) adults were included in the study subjects; (3) minimum sample size was < 20.

### 2.3. Data extraction

Two authors independently extracted data (FC and KW). The following information was extracted: first author, year of publication, country where the study was conducted, total number of people included in a study, six hemodynamic parameters, four respiratory parameters, intensive care unit (ICU) stay duration, length of hospital stay, two safety outcomes, etc. Any disagreements were settled by discussion until a consensus was reached or by consulting the third reviewer (YZZ).

### 2.4. Outcome measures

Outcome measures are as follows: (1) Hemodynamic parameters: RHC measures including mPAP, pulmonary artery systolic pressure (PASP), and PVR, pulmonary arterial/aortic pressure (PA/AO), systolic blood pressure (SBP), and heart rate (HR). (2) Respiratory parameters: blood oxygen saturation (SpO_2_), oxygenation index (OI): fraction of inspired oxygen × mean airway pressure/arterial oxygen pressure, partial pressure of arterial oxygen (PaO_2_), and mechanical ventilation duration. (3) Duration of intensive care unit (ICU) stay. (4) Length of hospital stay. (5) Safety outcomes: mortality and PH crisis. We extracted data on twelve continuous variables (mPAP, PASP, PVR, PA/AO, SBP, HR, OI, PaO_2_, SpO_2_, mechanical ventilation duration, duration of ICU stay, and length of hospital stay). Differences in the mean change from baseline for the experimental and control group, respectively, and standard deviation (SD) were calculated. We used median [range, size of a sample or the sample], median [interquartile range], and 95% CIs (*P*-value) when the mean and SD were not available, then converted them to mean and SD (31). In terms of dichotomic variables (mortality and PH crisis), the number of events (r) that occurred in participants and the number of participants (N) were extracted for each group. Trials comparing the effects of different treatment doses of the same type of PAH-targeted drugs and therapeutic duration were incorporated into the same group or the last node in the study.

### 2.5. Quality assessment

The quality of studies included in the meta-analysis was assessed by two investigators using the Cochrane risk of bias tool and the RevMan 5.3 software (32). Any disagreements on rating the quality of each trial were solved by a discussion with the third researcher (YYZhu). The following domains were considered for quality assessment: random sequence generation (selections bias), allocation concealment (selections bias), blinding of participants and personnel (performance bias), blinding of outcome assessment (detection bias), incomplete outcome data (attrition bias), selective reporting (reporting bias), and other bias. The overall risk of bias was rated as three levels: low risk of bias (all items were with low risk, or at least five items were with low risk and the remaining two unclear), unclear risk of bias (>2 items were with unclear risk), and high risk of bias (≥1 quality dimension suggested high bias) (33).

### 2.6. Network meta-analysis

A Bayesian network meta-analysis was performed using the R software (version 4.0.4, GeMTC package) used to call JAGS 4.3.0 (34). Continuous outcomes were presented as weighted mean differences (WMDs) with 95% confidence intervals (CIs). Risk ratios (RRs) with 95% confidence intervals (CIs) of were used as the effect indexes for dichotomous outcomes. The random-effects model (REM) of Markov Chain Monte Carlo (MCMC) was employed and four MCMC chains were adopted for simulation. The number of iterations was set to 50,000, with the first 20,000 iterations for annealing to remove the effect of the initial value and the last 30,000 iterations for sampling. Information criterion (DIC) values of consistency and inconsistency analyses were used to evaluate the convergence of models. The difference in DIC values between the two models was no more than five indicated good consistency between models. Therefore, the consistency analysis was adopted. The local inconsistency was examined by the node splitting method. Results with *P* < 0.05 suggested the existence of local inconsistency (35). Heterogeneity was quantified by *I*^2^ statistic. A random-effects model was adopted when overall heterogeneity was >50%. Otherwise, a fixed-effects model was adopted. To give a better explanation of RRs or WMDs, each intervention's probability was calculated by the surface under the cumulative ranking curves (SUCRA) (36). When a SUCRA value ranges from 0 to 1, a higher SUCRA value indicates a greater possibility that an intervention was among the top rank. Moreover, the effects between intervention groups were described using relative effect tables.

## 3. Results

**Figure 1:**
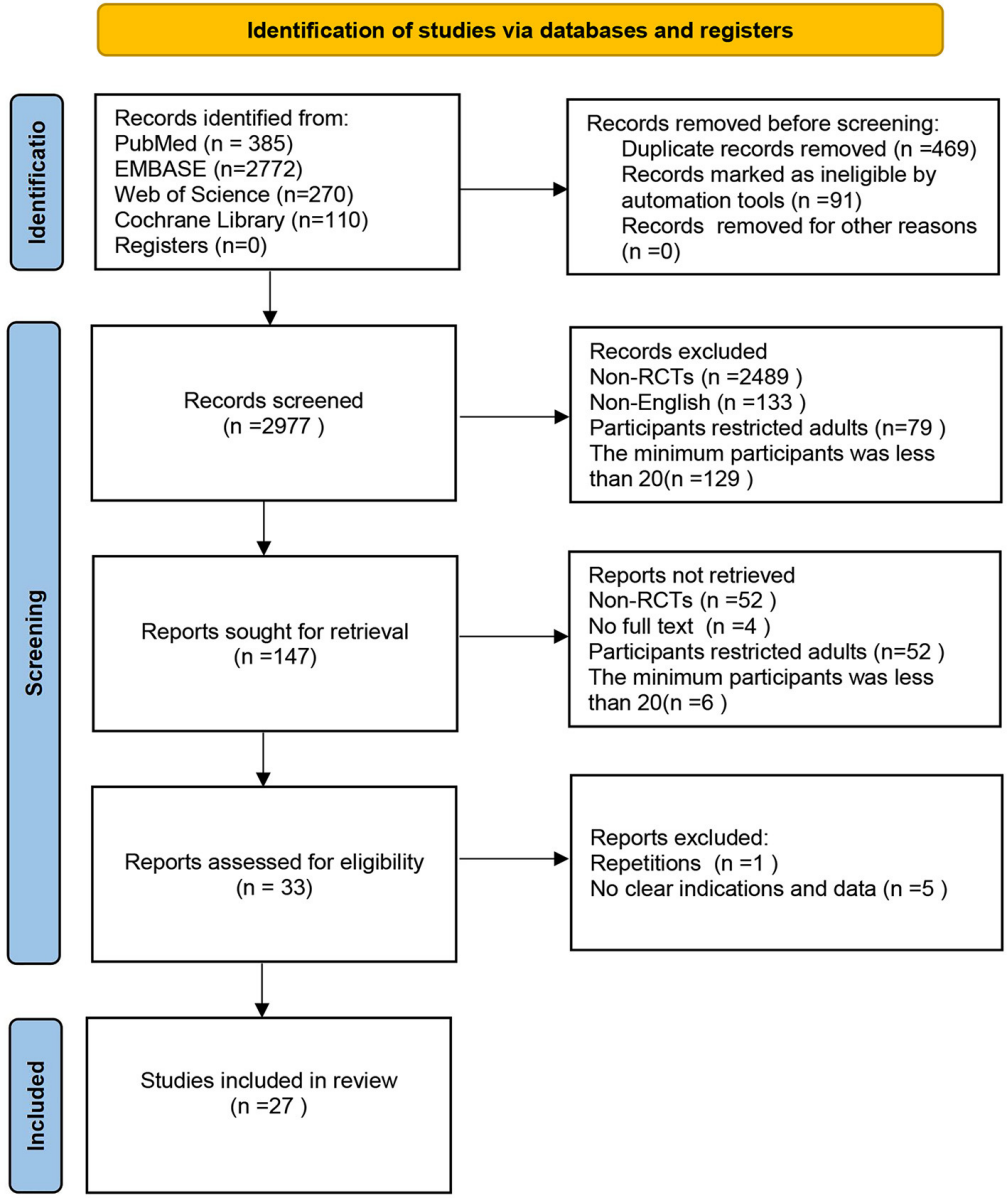
Flowchart for literature retrieval and the process of selection.

Literature search and screening yielded 3,537 articles and retrieved expressions are presented in Supplementary Table 1. A total of 560 duplicate publications were removed. Titles and abstracts of 2,830 articles were screened, and 147 articles were reviewed by reading the full text. Finally, 27 studies involving 719 pediatric subjects with PHA were included for a network meta-analysis. Literature screening strategy is shown in Figure 1. Baseline characteristics of eligible studies and basic information on subjects are presented in Table 1. Changes in mPAP, PASP, PVR, PA/AO, SBP, HR, SpO_2_, OI, and PaO_2_ are demonstrated in Supplementary Tables 2–10. Data on mechanical ventilation duration, length of ICU stay, hospital stay length, mortality, and PH crisis are shown in Supplementary Tables 11–15.

**Table 1:** Basic characteristics of the included trials.

**References**	**Study design**	**Country**	**Gender, *N* (Male/ Female)**	**Age (I/C)**	**Weight (I/C, kg)**	**Diagnosis**	**Intervention and dosages**	**Control**	**N(I/C)**	**Assessment time**	**Measurement method**
Namachivayam et al. (37)	SC	Australia	NR	0.5/0.3Y	4.6/4.0	Rebound PH	Sildenafil 0.4 mg/kg	Placebo	15/14	4 H	Catheter and UCG
Peiravian et al. (38)	SC	Iran	24/18	5.3/4.0 Y	14.3/12.9	POPH	Sildenafil 0.3 mg/kg q3h	Placebo	20/22	24 H	Catheter
Vargas-Origel et al. (39)	SC	Mexico	29/22	37.8/38.8 W	3.0/3.0	PPHN	Sildenafil 3 mg/kg q6h	Placebo	31/20	25 H	UCG
Uslu et al. (40)	SC	Turkey	36/29	38.5/38.3 W	3.2/3.3	PPHN	Sildenafil 0.5 mg/kg q6h	MgSO4	31/34	14 M	Catheter
Mohamed and Ismail (41)	SC	Saudi Arabia	26/21	39.7/38.8 W	3.5/3.5	PPHN	Bosentan 1 mg/kg bid	Placebo	24/23	6 M	UCG
Farah et al. (42)	SC	Iran	19/29	13.5/25.4/ 12.3 M	7.1/8.9/6.2	POPH	G1:Sildenafil 0.3 mg/kg q3h G2:Sildenafil 0.3 mg/kg q3h+ Milrinone 0.75 ug/kg/min	Milrinone 0.75 ug/kg/min	16/16/16	48 H	Catheter
Zhang et al. (43)	SC	Australia	32/28	3.2/2.9 Y	NR	POPH	IV PGE1 30 ng/kg/min qd or 100 μg nebulized PGE1 q8h	Captopril	40/20	10 D	UCG
Kahveci et al. et al. (44)	SC	Turkey	26/21	39.9/39.2 W	3.3/3.3	PPHN	Iioprost 1–2.5 ug/kg q2–4h	Sildenafil 0.5 mg/kg q6h	20/27	8 D	UCG
Giordano et al. (45)	SC	Italy	18/12	55/59 M	19.7/21.1	POPH	Sildenafil 0.35 mg/kg q4h	No drug use	13/17	72 H	Catheter
Sharma et al. (46)	SC	India	25/21	3.2/3.5 Y	12.6/12.8	POPH	Sildenafil 0.5 mg/kg bid	Placebo	23/23	16 W	Catheter
Al Omar et al. (47)	SC	Qatar	15/9	38.1/39 W	3.1/3.2	PPHN	Sildenafil 2 mg/kg q6h	Placebo	13/11	14 D	UCG
Onan et al. (48)	SC	Turkey	13/14	7.8/5.8 M	5.2/5.6	POPH	Iloprost 2.0 ng/kg/min	Placebo	15/12	30 D	Catheters
Steinhorn et al. (49)	MC	Washington, DC	6/15	39.2/38.6 W	3.4/3.2	PPHN	Bosentan 2 mg/kg bid	Placebo	13/8	12 M	UCG
Bhasin et al. (50)	SC	India	28/32	14.5/15.8 M	9.0/9.0	POPH	Pre- and post-operative Sildenafil 0.5 mg/kg q6h	Post-operative Sildenafil 0.5 mg/kg q6h	30/30	15 D	Catheters
Bigdelian and Sedighi (51)	SC	Iran	39/24	5.4/5.7/5.4 M	6.6/6.8/6.4	POPH	G1: Pre- and post-operative Sildenafil 0.3 mg/kg q4h G2: post-operative Sildenafil 0.3 mg/kg q4h	No drug use	22/21/20	48 H	Catheters
El-Ghandour et al. (52)	SC	Egypt	42/18	NR	3.0/3.0/3.0	PPHN	G1: Sildenafil 0.5 mg/kg q6h G2: Sildenafil 0.5 mg/kg q6h +Milrinone 0.5 ug/kg/min	Milrinone 0.5 ug/kg/min	20/20/20	14 d	UCG
Huang et al. (53)	SC	China	44/35	3.6/3.5 M	5.6/5.8	POPH	Teprostinil 1.25 ng/kg/min	Milrinone 0.5–0.75 ug/kg/min	36/43	NR	UCG
Patel et al. (54)	SC	India	17/13	12/12 M	6/6.8	POPH	Pre- and post-operative Sildenafil 0.5 mg/kg q6h	Post-operative Sildenafil 0.5 mg/kg q6h	15/15	NR	UCG
William et al. (55)	SC	Egypt	100/67	2.9/3.2 Y	10.1/11.8	PPAH	Sildenafil < 1 year 0.5–1 mg/kg >1 year and < 20 kg 10 mg tid >1 year and >20 kg 20 mg tid	Conventional therapy	57/110	12 W	UCG
Pierce et al. (56)	MC	United Kingdom	33/26	41.2/46.3 H	3.3/3.4	PPHN	IV Sildenafil 0.1 mg/kg, over 30 min; maintenance: 0.03 mg/kg/h	Placebo	29/30	14 D	UCG
Thandaveshwara et al. (57)	SC	India	12/11	8/8 H	3/3	PPHN	Sildenafil 0.48 mg/kg q6h	Placebo	11/12	72 H	UCG
Barst et al. (21)	MC	New York	89/145	1–17 Y	17/17	PPAH	Sildenafil Targeted concentration^a^	Placebo	165/69	16 W	Catheter
Takahashi et al. (58)	SC	Japan	13/7	4.6/11.0 Y	NR	POPH	Beraprost Beginning with 1 ug/kg	Placebo	7/13	15 M	Catheters
Fatima et al. (59)	SC	Pakistan	55/45	3.7/3.4 D	NR	PPHN	Sildenafil 2 mg/kg tid+ Bosentan 1 mg/kg bid	Sildenafil 2 mg/kg tid	50/50	72 H	UCG
Farhangdoust et al. (60)	SC	Iran	28/12	33.5/33.5 W	2.2/2.1	PPHN	Bosentan 1 mg/kg q12h	Sildenafil 0.4 mg/kg q12h	15/25	12 D	UCG
Bagheri et al. (61)	SC	Iran	NR	37.5/37.5 W	2.7/2.7	PPHN	Sildenafil 3 mg/kg/day + Milrinone	Milrinone	40/40	72 H	UCG
Xu et al. (62)	SC	American	17/5	28.0/12.8 M	9.2/5.9	POPH	Iloprost 30 or 50 ng/kg/min	Placebo	15/7	< 30 D	UCG

MC, many centers; SC, single-center; NR, not reported.

N, number; I, Intervention group; C, control group; Y, years; M, months; W, weeks; D, days; H, hours; q2–4 h every 2–4 h, q3h every 3h, q4h every 4 h, q6h every 6 h, q8h every 8 h, q12h every 12 h.

UCG, ultrasonic cardiogram, Rebound; PH, rebound pulmonary hypertension; PPHN, persistent pulmonary hypertension of the newborn; POPH, pediatric post-operative pulmonary hypertension; PPAH, pediatric pulmonary artery hypertension.

^a^Targeted concentration: oral sildenafil achieve maximum plasma concentrations of 47, 140, and 373 ng/ml, respectively.

### 3.1. Statistical analysis

**Figure 2:**
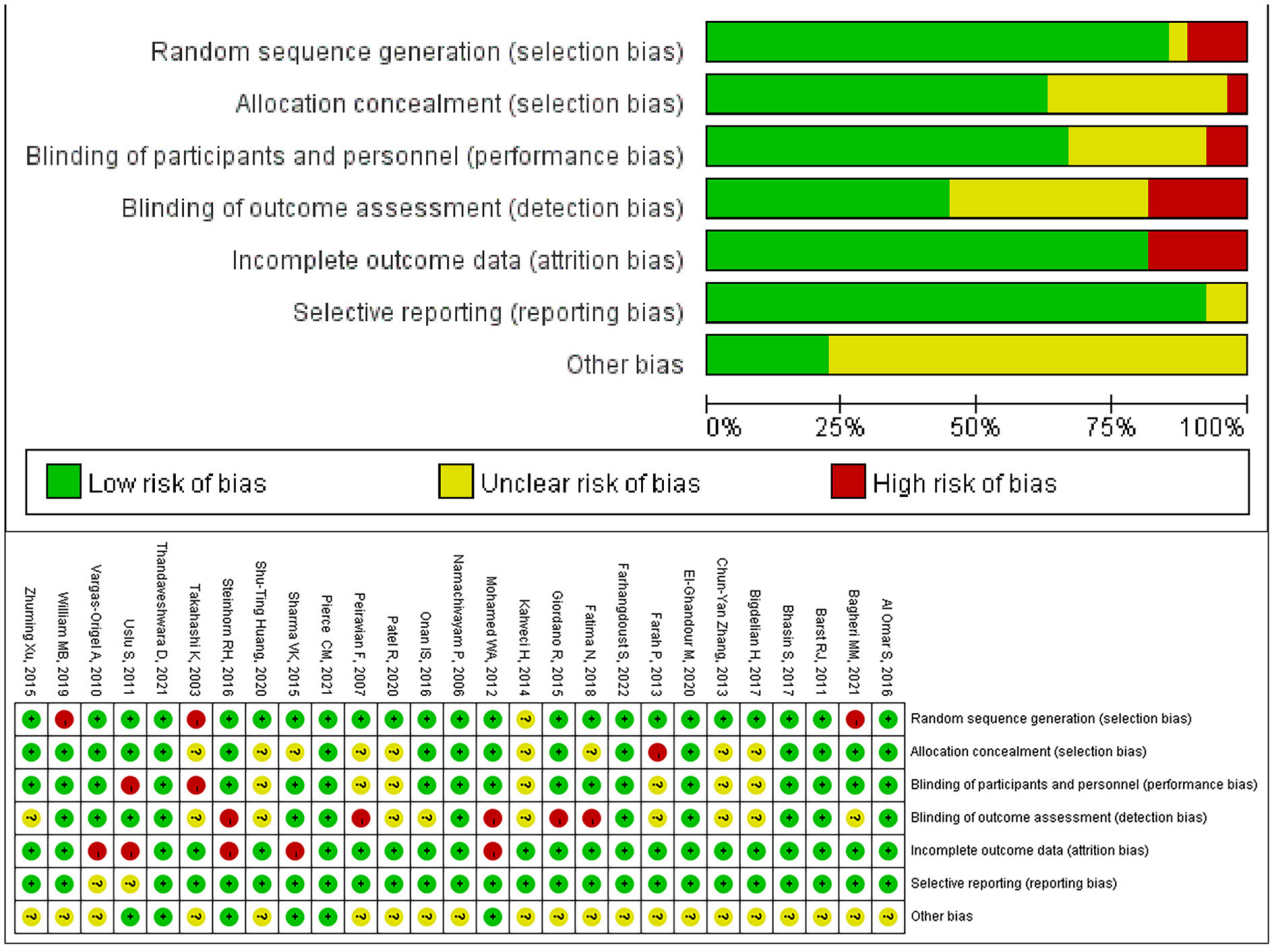
Risk of bias.

**Figure 3:**
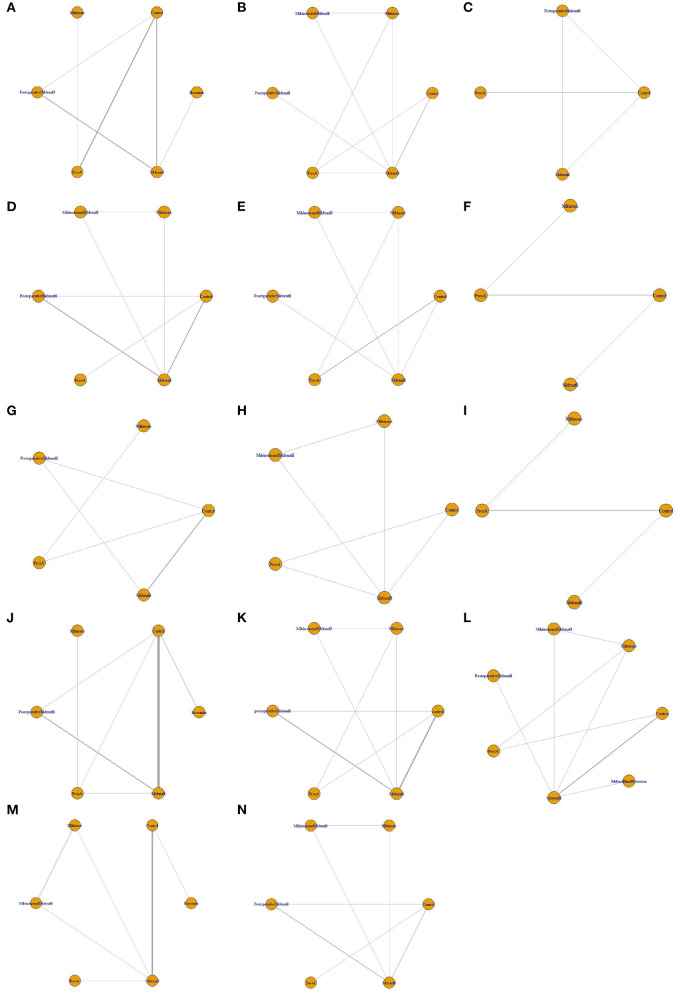
Network diagrams of fourteen outcomes of different interventions. **(A)** Mean pulmonary artery pressure (mPAP) change; **(B)** pulmonary artery systolic pressure (PASP) change; **(C)** pulmonary vascular resistance (PVR) change; **(D)** pulmonary arterial/aortic pressure (PA/AO) change; **(E)** systolic blood pressure (SBP) change; **(F)** heart rate (HR) change; **(G)** blood oxygen saturation (SpO_2_) change; **(H)** oxygenation index (OI) change; **(I)** partial pressure of arterial oxygen (PaO_2_) change; **(J)** mechanical ventilation duration; **(K)** intensive care unit stay (ICU) duration; **(L)** Hospital stay duration; **(M)** mortality; **(N)** pulmonary hypertension (PH) crisis. The network plots show how a comparison of different intervetions. Each vertex represents a kind of intervention. The thickness of the straight line represents the number of trials compared.

The results of the risk of bias assessment for included studies are presented in Figure 2. Network diagrams involving comparisons of different treatments regarding seven outcome measures are presented in Figure 3. The node-splitting analysis results are summarized in Supplementary Table 16.

### 3.2. Hemodynamic parameters

#### 3.2.1. mPAP

WMDs with 95% CIs for mPAP from the network meta-analysis are displayed in Supplementary Table 17. The overall consistency was 9%, so a fixed-effects model was adopted for data analysis. SUCRA values for mPAP are shown in Table 2 and Supplementary Figure 1. Twelve included studies with 799 patients reported mPAP. There was no statistically significant difference between ERAs, PDE-5i, and ProA for lowering mPAP [bosentan vs. sildenafil: WMD = −3.29, 95% CI (−21.67 ~ 14.99); sildenafil vs. ProsA: WMD = −0.62, 95% CI (−14.94 ~ 12.17); bosentan vs. ProsA: WMD = −2.69, 95% CI (−24.72 ~ 20.82)]. According to the SUCRA values, PHA-targeted drugs for lowering mPAP can be ranked as follows: bosentan (SUCRA 70.8%) >ProsA (SUCRA 63.5%) >sildenafil (SUCRA 60.6%). The results revealed that ERAs were likely to be the best option for reducing mPAP.

**Table 2:** Surface under the cumulative ranking curve (SUCRA) results for outcome measures.

**Treatment**	**mPAP**	**PASP**	**PVR**	**PA/AO**	**SBP**	**HR**	**OS**	**OI**	**PaO_2_**	**mechanical**	**ICU**	**Hospital**	**Mortality**	**PH**
	**Change**	**Change**	**Change**	**Change**	**Change**	**Change**	**Change**	**Change**	**Change**	**ventilation**	**stay**	**stay**		**crisis**
Bosentan	0.708	–	–	–	–	–	–	–	–	0.995	–	–	0.774	–
ProsA	0.635	0.807	0.702	0.296	0.351	0.816	0.442	0.708	0.798	0.778	0.984	0.772	0.474	0.068
Sildenafil	0.606	0.43	0.523	0.568	0.572	0.542	0.841	0.53	0.516	0.375	0.547	0.324	0.498	0.517
Post	0.423	0.22	0.497	0.184	0.248	–	0.546	–	–	0.2	0.29	0.182	–	0.633
Sildenafil														
Milrinone	0.472	0.494	–	0.794	0.699	0.142	0.358	0.567	0.153	0.618	0.774	0.642	0.411	0.995
Control	0.155	0.105	0.279	0.226	0.662	0.501	0.313	0.592	0.533	0.033	0.152	0.53	0.198	0.134
Milrinone and Sildenafil	–	0.943	–	0.932	0.467	–	–	0.104	–	–	0.253	0.344	0.645	0.654
Sildenafil and Bosentan	–	–	–	–	–	–	–	–	–	–	–	0.706	–	

#### 3.2.2. PASP

WMDs with 95% CIs for PASP from network meta-analysis are displayed in Supplementary Table 18. The overall consistency was 0%, so a fixed-effects model was adopted for data analysis. SUCRA values for PASP are demonstrated in Table 2 and Supplementary Figure 1. Seven studies with 339 patients contributed to the analysis of PASP. No significant difference was found between PDE-5i and ProA for decreasing PASP [sildenafil vs. ProsA: WMD=-1.48, 95% CI (−17.43 ~ 11.87)]. Based on SUCRA values, targeted drugs for decreasing PASP can be ranked as follows: Milrinone and sildenafil (SUCRA 94.3%) > ProsA (SUCRA 80.7%) > Milrinone (SUCRA 49.4%) > sildenafil (SUCRA 43.0%). The results showed that ProA was possibly superior to PDE-5i for decreasing PASP.

#### 3.2.3. PVR

WMDs with 95% CIs for PVR from network meta-analysis are displayed in Supplementary Table 19. The overall consistency was 18%, so a fixed-effects model was adopted for data analysis. SUCRA values for PVR are shown in Table 2 and Supplementary Figure 1. In light of PVR, three studies with 105 patients were analyzed. There was no statistically significant difference between PDE-5i and ProA for moderating PVR [sildenafil vs. ProsA: WMD = −1.42, 95% CI (−17.40 ~ 11.60)]. SUCRA value rankings among treatments regarding moderating PVR are as follows: ProsA ranked first (SUCRA 70.2%), followed by sildenafil (SUCRA 52.3%). The results showed that ProA was probably superior to PDE-5i for moderating PVR.

#### 3.2.4. PA/AO

WMDs with 95% CIs for PA/AO from network meta-analysis are presented in Supplementary Table 20. The overall consistency was 0%, so a fixed-effects model was adopted for data analysis. SUCRA values for PA/AO are demonstrated in Table 2 and Supplementary Figure 1. Seven studies with 300 patients reported PA/AO. There was no statistically significant difference between PDE-5i and ProA in reducing the ratio of PA/AO [sildenafil vs. ProsA: WMD = 0.08, 95% CI (−0.15 ~ 0.33)]. Interestingly, the ratio of PA/AO was significantly reduced in Milrinone and sildenafil [WMD = 0.26, 95% CI (0.04 ~ 0.50)] vs. placebo. According to the SUCRA values, targeted drugs for reducing the ratio of PA/AO can be ranked as follows: Milrinone and sildenafil (SUCRA 93.2%) > Milrinone (SUCRA 79.4%) > sildenafil (SUCRA 56.8%) > ProsA (SUCRA 29.6%). The results showed that PDE-5i was presumably superior to ProA for lowering the ratio of PA/AO.

#### 3.2.5. SBP

WMDs with 95% CIs for SBP from network meta-analysis are displayed in Supplementary Table 21. The overall consistency was 0%, so a fixed-effects model was adopted for data analysis. SUCRA values for SBP are shown in Table 2 and Supplementary Figure 1. Six included studies with 428 patients reported on SBP. No statistically significant difference emerged between PDE-5i and ProA for affecting SBP [sildenafil vs. ProsA: WMD = −2.06, 95% CI (−12.58 ~ 8.15)]. The ranking of SUCRA values for targeted drugs regarding reducing the occurrence of hypotension is as follows: Milrinone (SUCRA69.9%) > sildenafil (SUCRA 57.2%) > Milrinone and sildenafil (SUCRA 46.7%) > ProsA (SUCRA 35.1%). The results showed that PDE-5i was possibly superior to ProA for reducing the occurrence of hypotension.

#### 3.2.6. HR

WMDs with 95% CIs for HR from network meta-analysis are displayed in Supplementary Table 22. The overall consistency was 4%, so a fixed-effects model was adopted for data analysis. SUCRA values for HR are demonstrated in Table 2 and Supplementary Figure 1. Four included studies with 308 patients reported on HR. There was no statistically significant difference between PDE-5i and ProA for lowering HR, sildenafil vs. ProsA [WMD = −4.078, 95% CI (−24.40 ~ 16.19)]. According to the SUCRA, ProsA ranked first among all the treatments (SUCRA 81.6%) followed by sildenafil (SUCRA 54.2%). The results showed that ProA was potentially superior to PDE-5i for lowering HR.

### 3.3. Respiratory parameters

#### 3.3.1. SpO_2_

WMDs with 95% CIs for SpO_2_ from network meta-analysis are displayed in Supplementary Table 23. The overall consistency was 7%, so a fixed-effects model was adopted for data analysis. SUCRA values for SpO_2_ are shown in Table 2 and Supplementary Figure 1. Five studies involving 379 patients contributed to the analysis of SpO_2_. No statistically significant difference emerged between PDE-5i and ProA for increasing SpO_2_ [sildenafil vs. ProsA: WMD = −1.06, 95% CI (−4.39 ~ 2.04)]. Based on the SUCRA value rankings, targeted drugs for increasing SpO_2_ can be presented in descending order: sildenafil (SUCRA 84.1%) > Post-operative sildenafil (SUCRA 54.6%) > ProsA (SUCRA 44.2%). The results showed that PDE-5i was possibly superior to ProA for increasing SpO_2_.

#### 3.3.2. OI

WMDs with 95% CIs for OI from network meta-analysis are displayed in Supplementary Table 24. The overall consistency was 6%, so a fixed-effects model was adopted for data analysis. SUCRA values for OI are demonstrated in Table 2 and Supplementary Figure 1. Four studies with 193 patients investigated OI. Data analysis revealed no statistically significant difference between PDE-5i and ProA for improving OI [sildenafil vs. ProsA: WMD = 1.21, 95% CI (−4.75 ~ 7.32)]. Based on the SUCRA, targeted drugs for improving OI can be ranked as follows: ProsA (SUCRA 70.8%) > Milrinone (SUCRA 56.7%) > sildenafil (SUCRA 53.0%). The results showed that ProA was presumably superior to PDE-5i for improving OI.

#### 3.3.3. PaO_2_

WMDs with 95% CIs for PaO_2_ from network meta-analysis are displayed in Supplementary Table 25. The overall consistency was 15%, so a fixed-effects model was adopted for data analysis. SUCRA values for PaO_2_ are shown in Table 2 and Supplementary Figure 1. A total of four studies with 175 patients reported PaO_2_. No statistically significant difference was observed between PDE-5i and ProA for increasing PaO_2_ [sildenafil vs. ProsA: WMD = 8.07, 95% CI (−41.91 ~ 59.02)]. Based on the SUCRA values, targeted drugs for increasing PaO_2_ can be ranked as follows: ProsA (SUCRA 79.8%) > sildenafil (SUCRA 51.6%). The study showed that ProA was possibly superior to PDE-5i for increasing PaO_2_.

#### 3.3.4. Duration of mechanical ventilation

WMDs with 95% CIs for the duration of mechanical ventilation from network meta-analysis are displayed in Supplementary Table 26. The overall consistency was 35%, so a fixed-effects model was adopted for data analysis. SUCRA values for the duration of ventilation are demonstrated in Table 2 and Supplementary Figure 1. For the duration of ventilation, a sum of sixteen studies with 684 patients was included for analysis. Compared with the placebo, a significantly shorter duration of mechanical ventilation was observed in patients to whom bosentan [WMD = −166.15, 95% CI (−182.00–−118.81)], sildenafil [WMD = 9.04, 95% CI (1.32 ~ 20.42)], or ProsA [WMD = 87.09, 95% CI (35.36 ~ 137.52)] were administered. Meanwhile, there were statistically significant differences between ERAs, PDE-5i, and ProA for shortening the duration of mechanical ventilation [bosentan vs. sildenafil: WMD = −157.04, 95% CI (−173.88 ~ −106.11); sildenafil vs. ProsA: WMD = −77.68, 95% CI (−127.69 ~ −25.85)]; bosentan vs. ProsA: WMD = −77.61, 95% CI (−130.41 ~ −15.51)]. Based on the SUCRA values, targeted drugs for shortening the duration of ventilation can be ranked as follows: bosentan (SUCRA 99.5%) > ProsA (SUCRA 77.8%) > Milrinone (SUCRA61.8%) > sildenafil (SUCRA37.5%). ERAs have the highest probability of being the best therapy choices for shortening the duration of ventilation.

### 3.4. Length of stay in ICU

WMDs with 95% CIs for ICU length of stay from network meta-analysis are displayed in Supplementary Table 27. The overall consistency was 16%, so a fixed-effects model was adopted for data analysis. SUCRA values for the time of ICU stay are shown in Table 2 and Supplementary Figure 1. Ten studies with 454 patients contributed to the analysis of length of ICU stay. The results showed that sildenafil [WMD = 18.63, 95% CI (3.22 ~ 39.70)] and ProsA [WMD = 86.32, 95% CI (27.25 ~ 138.31)] were significantly superior to the control group. Meanwhile, there was a statistically significant difference between PDE-5i and ProA for lessening the time of ICU stay, sildenafil vs. ProsA (WMD, −67.35, 95% CI, −117.17 ~ −6.91). According to the SUCRA, targeted drugs for reducing the length of stay in ICU can be ranked as follows: ProsA (SUCRA 98.4%) > Milrinone (SUCRA 77.4%) > sildenafil (SUCRA 54.7%). The study showed that ProA was possibly superior to PDE-5i for shortening the length of stay in the ICU.

### 3.5. Length of hospital stay

WMDs with 95% CIs for the length of stay in hospitals from network meta-analysis are displayed in Supplementary Table 28. The overall consistency was 4%, so a fixed-effects model was adopted for data analysis. SUCRA values for the length of hospital stay are demonstrated in Table 2 and Supplementary Figure 1. Nine studies with 540 patients contribute to the hospital length of stay. It was found that there were no statistically significant differences between ERAs, PDE-5i, and ProA for shortening the length of hospital stay [sildenafil and bosentan vs. sildenafil: WMD=2.54, 95% CI (−3.30–8.35); sildenafil vs. ProsA: WMD = −3.08, 95% CI (−8.80 ~ 3.40); sildenafil and bosentan vs. ProsA: WMD = −0.55, 95% CI (−8.62 ~ 8.27)]. Based on SUCRA values, targeted drugs for increasing PaO_2_ can be ranked as follows: ProsA (SUCRA 77.2%) > sildenafil and bosentan (SUCRA 70.6%) > Milrinone (SUCRA 64.2%) > sildenafil (SUCRA 32.4%). The results showed that ProA was possibly superior to PDE-5i for shortening the length of stay in hospital. At the same time, ProA was conceivably superior to PDE-5i combined with ERAs for shortening the length of stay in the hospital.

### 3.6. Safety outcomes

#### 3.6.1. Mortality

RRs with 95% CIs for mortality from network meta-analysis are displayed in Supplementary Table 29. The overall consistency was 6%, so a fixed-effects model was adopted for data analysis. SUCRA values for mortality are shown in Table 2 and Supplementary Figure 1. A total of ten studies with 619 patients evaluated mortality. The analysis showed that there was no statistically significant difference between ERAs, PDE-5i, and ProA for decreasing mortality: bosentan vs. sildenafil [RR = −1.41, 95% CI (−5.51 ~ 2.20)]; sildenafil vs. ProsA [RR = −5.40, 95% CI (−2.87 ~ 2.80)]; bosentan vs. ProsA [RR = −1.43, 95% CI (−6.30 ~ 3.21)]. SUCRA value rankings for targeted drugs regarding decreasing mortality are as follows: bosentan (SUCRA 77.4%) > Milrinone and sildenafil (SUCRA 64.5%) > sildenafil (SUCRA 49.8%) > ProsA (SUCRA 47.4%). ERAs have the highest probability of being the effective PHA-targeted drug for decreasing mortality.

#### 3.6.2. PH crisis

RRs with 95% CIs for PH crisis from network meta-analysis are displayed in Supplementary Table 30. The overall consistency was 0%, so a fixed-effects model was adopted for data analysis. SUCRA values for the PH crisis are demonstrated in Table 2 and Supplementary Figure 1. Five included studies with 240 patients investigated the PH crisis. The network meta-analysis results showed that compared with the placebo, the occurrence of PH crisis was significantly reduced in patients taking milrinone and sildenafil [RR = 27.25, 95% CI (4.56 ~ 81.92)], post-operative sildenafil [RR = 27.12, 95% CI (4.52 ~ 81.77)], or sildenafil [RR = 26.05, 95% CI (3.98 ~ 80.68)]. Meanwhile, there was a statistically significant difference between PDE-5i and ProA for decreasing PH crisis: ProsA vs. Milrinone and sildenafil [RR = −28.02, 95% CI (−82.58 ~ −4.73)]; ProsA vs. Post-operative sildenafil [RR = −27.96, 95% CI (−82.50 ~ −4.68)]; ProsA vs. sildenafil [RR = 26.93, 95% CI (4.15 ~ 81.39)]. Based on SUCRA values, targeted drugs for decreasing the PH crisis can be ranked as follows: Milrinone and sildenafil (SUCRA 65.4%) > Post-operative sildenafil (SUCRA 63.3%) > sildenafil (SUCRA 51.7%) > ProsA (SUCRA 6.8%). The results showed that PDE-5i was possibly superior to ProA for decreasing the PH crisis. In addition, we found that the combination of milrinone and sildenafil can prevent recurrent PHA, so the combination of them may be better than the use of sildenafil alone in reducing the PH crisis.

## 4. Discussion

This is the first network meta-analysis evaluating the efficacy and safety of three kinds of PAH-targeted agents (ERAs, PDE-5i, and ProsA) for the management of pediatric PAH. The clinical benefits of PAH-targeted agents for treating children with PAH were investigated, including reducing mechanical ventilation duration, shortening the length of ICU stay, and easing the PH crisis.

Meta-analysis results revealed no statistically significant difference concerning hemodynamic parameters among patients using ERAs, PDE-5i, or ProA. In terms of respiratory parameters, no statistically significant differences were found in SpO_2_, OI, and PaO_2_ among patients using ERAs, PDE-5i, or ProA, which differs from previous studies in which significant differences emerged regarding SpO_2_, HR, mPAP, and PVR with prolonged use of bosentan for PAH patients, compared with the basic hemodynamics (63–65). A non-network meta-analysis performed by Shu et al. showed that pulmonary vasodilators significantly decreased mPAP, OI, PA/AO, and PaO_2_, compared with the control group (14), which may be because it investigated the absolute value instead of differences in the mean change from baseline (mean change) of the parameters. The possible reasons responsible for this analysis results are as follows: (1) The majority of the included studies reported the absolute value, while the mean change from the baseline (mean change) of the parameters was estimated for this meta-analysis. (2) The uncertain effects of PAH-targeted medications on improving clinical outcomes regarding hemodynamic and respiratory parameters are possibly attributed to the heterogeneity between included studies. (3) The heterogeneity may be attributed to small sample sizes and the short duration of follow-up in the included studies. Nonetheless, bosentan, sildenafil, and ProsA significantly reduced mechanical ventilation duration, compared with the placebo. Sildenafil and ProsA significantly shortened the length of stay in the ICU, compared with the control group, which is in line with previous meta-analyses of RCTs (14). A possible explanation is that pulmonary vasodilators (ERAs, PDE-5i, and ProsA) can reduce the incidence of ventilator-related problems, such as barometric injury, ventilator-related pneumonia, and difficulty in removing the ventilation machine (66, 67), thus reducing the duration of mechanical ventilation and ICU stay. The most interesting finding is that bosentan significantly reduced mechanical ventilation duration compared with ProsA, while ProsA significantly reduced it compared with sildenafil. This finding is consistent with that of the study conducted by Kahveci et al. which revealed that longer duration of mechanical ventilation was observed in the sildenafil group than in the iloprost group (44), indicating that ProsA was better than sildenafil for treating PHA in terms of OI, PaO_2_ and other respiratory parameters. Another important finding is that ProsA significantly shortened the length of stay in the ICU compared with sildenafil. As for the length of hospital stay, differences between the ERAs, the PDE-5i, and the ProA group were not significant. This accords with earlier research demonstrating differences between the pediatric PAH and the control group were of no statistical significance considering the length of hospital stay (66). It is known that PAH is often associated with heart failure, respiratory failure, PH crisis, and other serious diseases (68). Thus, PAH-targeted medications alone cannot effectively shorten the length of hospital stay.

In terms of the safety outcomes, a previous meta-analysis illustrated that the estimated 3-, 6-month, 1-, and 2-year event-free survival rates among PAH patients were 96.6, 92.3, 62.6, and 39.6%, respectively, and 12 patients with PAH treated with aerosolized iloprost died during the follow-up period with a low mortality rate of 3.24% (69). Our finding is similar to the result of the meta-analysis conducted by Shu et al. (14) and Chen et al. (70) which demonstrated that mortality in children treated with ERAs and ProA was not significantly different from that in the control group. However, a meta-analysis conducted by Zhang et al. (66) showed that compared with the control group, mortality in the sildenafil group decreased, but the difference was not statistically significant between low- and high-dose sildenafil groups. A recent meta-analysis also revealed that pulmonary vasodilators decreased mortality significantly (14). However, several studies found that PAH-targeted drugs appeared to have no effects on mortality (71–73), which may be related to the small number of participants and a short duration of follow-up in the included trials. Thus, RCTs with large sample sizes are required. We found that sildenafil reduced the occurrence of PH crisis markedly compared with placebo and ProsA, which does not support previous research that revealed no significant differences in the incidence of PH crisis between the pediatric PAH and the control group (66). Interestingly, this network meta-analysis discovered that a combination of milrinone and sildenafil was associated with a significantly lower risk of PH, compared with the use of sildenafil alone, and the possible reason is that the occurrence of rebound PAH can be prevented by a combination of these two drugs.

## 5. Conclusion

The present network meta-analysis showed that PAH-targeted agents shortened the duration of mechanical ventilation and ICU stay and reduced PH crisis in pediatric patients with PAH. ERAs were more effective than ProsA for shortening the duration of mechanical ventilation. ProsA was associated with significantly shorter duration of mechanical ventilation and ICU stay, compared with PDE-5i. But PDE-5i was more effective considering reducing the incidence of PH crisis compared with ProsA. These findings help clinicians with medical decision-making. However, it is worth emphasizing that the choice of treatment should be based on both the feature of each therapy and individual characteristics. In addition, RCTs with larger sample sizes are required for further investigation.

## 6. Limitations

The present study has some limitations. First, a limited number of RCTs were included for a network meta-analysis. Therefore, subgroup analysis was not performed according to different PAH classifications. This study did not demonstrate the advantages and disadvantages of three kinds of PAH-targeted drugs based on the premise of controlling multiple influencing factors, for example, time of use, patient's age, PAH grades, and other factors. Thus, caution is needed when interpreting the findings of this study. Second, the diagnosis of PAH in included studies was based on ultrasonic cardiogram instead of cardiac catheterization. The reliability of results may be undermined considering that ultrasonic cardiogram is not the golden tool for screening PAH. Third, only four included studies investigated bosentan, so the results of this network meta-analysis are not comprehensive. Fourth, some parameters have not been assessed in an overwhelming majority of included trials, such as PVR, HR, OI, and PaO_2_, possibly leading to biased results. Fifth, some trials adopted a combination of targeted drugs for PAH with other traditional treatments such as milrinone, which may also affect the reliability of results. Beyond that, the effects of different ages on the response to PAH-targeted medications may influence the accuracy of conclusions. Lastly, more RCTs with high quality evaluating the long-term efficacy and safety of ERAs, PDE-5i, and ProA in pediatric PAH are required in the future considering the short duration of follow-up in included trials in the present study.

## Data availability statement

The original contributions presented in the study are included in the article/Supplementary material, further inquiries can be directed to the corresponding authors.

## Author contributions

Y-zZ and Z-wB conceived and designed the study. FC, KW, Y-zZ, and J-jJ retrieved relevant articles, extracted the data, analyzed the data, and wrote the manuscript. GZ, JL, PX, YT, SZ, WZ, and FH organized all tables and graphs. FC, KW, Y-zZ, and GZ checked and reviewed the article's final version. All authors approved the results and conclusions of this article.
