# Supplementary material for: Efficacy and safety of endothelin receptor antagonists, phosphodiesterase type 5 Inhibitors, and prostaglandins in pediatric pulmonary arterial hypertension: A network meta-analysis
Source: Front Cardiovasc Med. 2023 Jan 11;9:1055897. doi: 10.3389/fcvm.2022.1055897 (PMC9875131; doi:10.3389/fcvm.2022.1055897)
Supplement: Supplementary file 1 [file Data_Sheet_1.PDF]

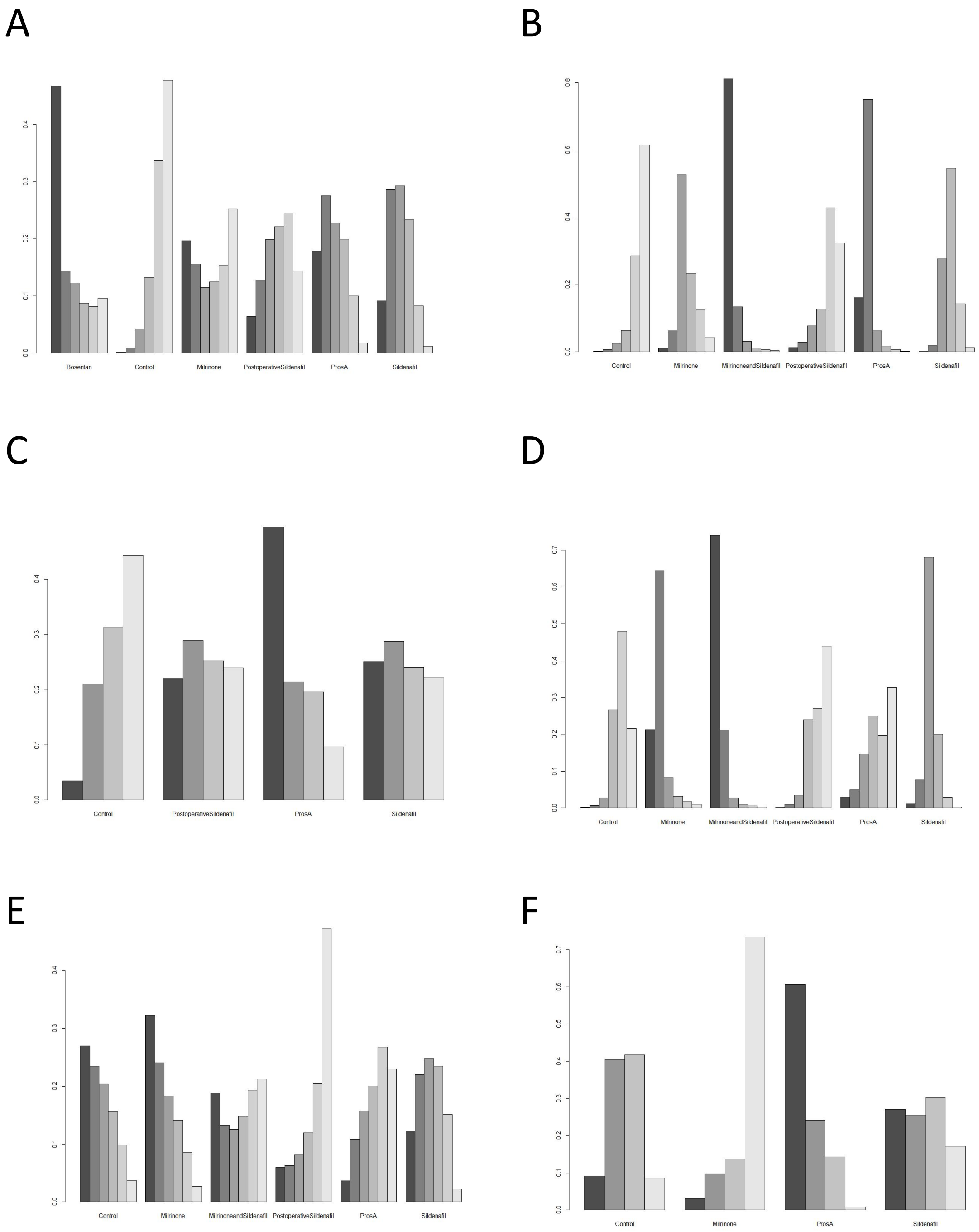
A
Bosentan
Control
Milrinone
PostoperativeSildenafil
ProsA
Sildenafil
B
Control
Milrinone
MilrinoneandSildenafil
PostoperativeSildenafil
ProsA
Sildenafil
C
Control
PostoperativeSildenafil
ProsA
Sildenafil
D
Control
Milrinone
MilrinoneandSildenafil
PostoperativeSildenafil
ProsA
Sildenafil
E
Control
Milrinone
MilrinoneandSildenafil
PostoperativeSildenafil
ProsA
Sildenafil
F
Control
Milrinone
ProsA
Sildenafil

G

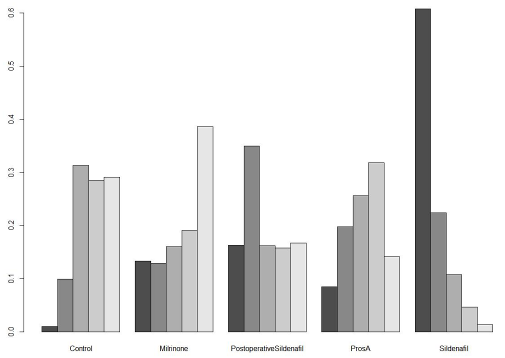
0.0 0.1 0.2 0.3 0.4 0.5 0.6
Control
Milrinone
PostoperativeSildenafil
ProsA
Sildenafil

H

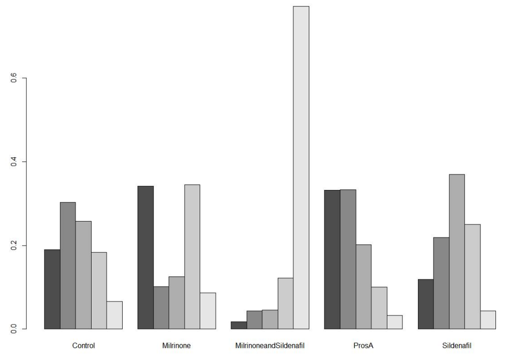
0.0 0.2 0.4 0.6
Control
Milrinone
MilrinoneandSildenafil
ProsA
Sildenafil

I

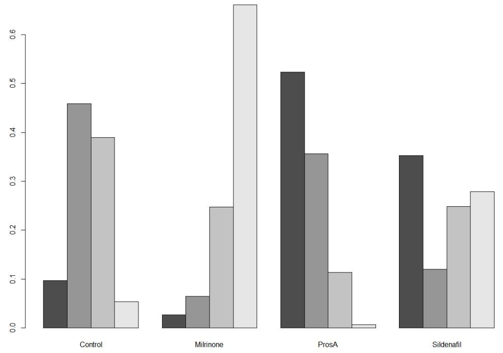
0.0 0.1 0.2 0.3 0.4 0.5 0.6
Control
Milrinone
ProsA
Sildenafil

J

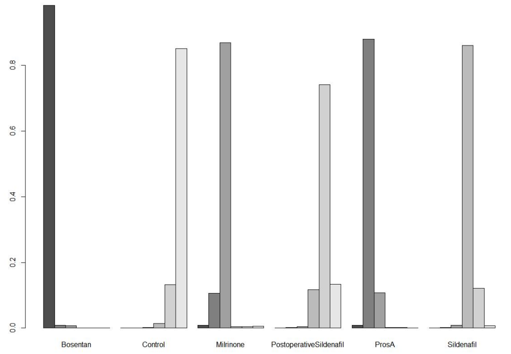
0.0 0.2 0.4 0.6 0.8
Bosentan
Control
Milrinone
PostoperativeSildenafil
ProsA
Sildenafil

K

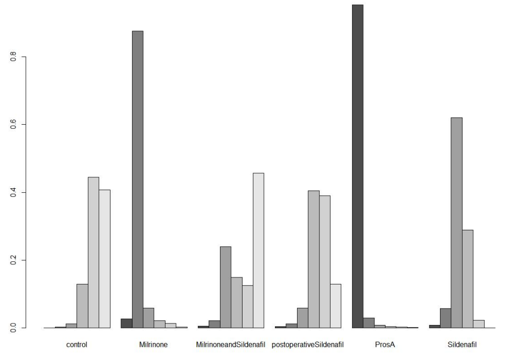
0.0 0.2 0.4 0.6 0.8
control
Milrinone
MilrinoneandSildenafil
postoperativeSildenafil
ProsA
Sildenafil

L

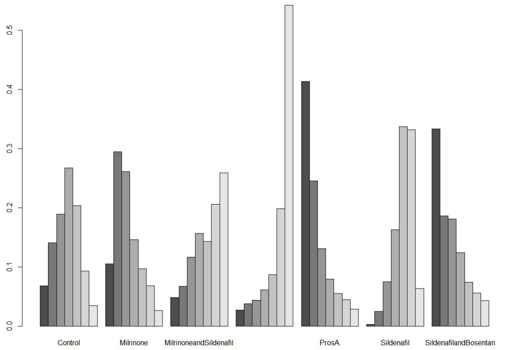
0.0 0.1 0.2 0.3 0.4 0.5
Control
Milrinone
MilrinoneandSildenafil
ProsA
Sildenafil
SildenafilandBosentan

M

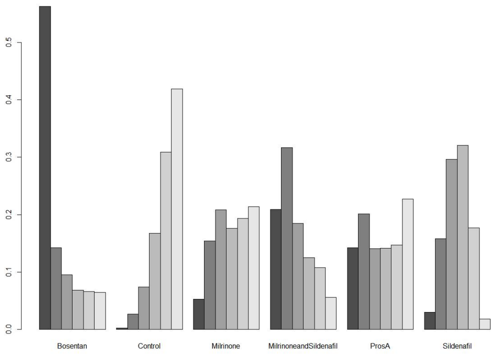

N

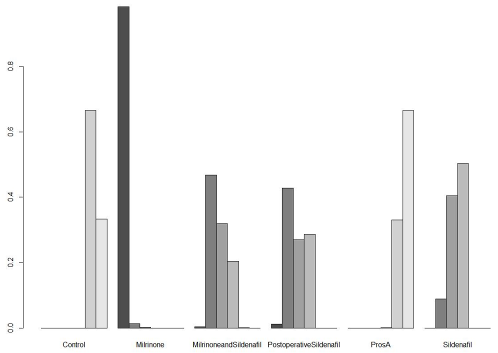

Supplementary Figure 1. Probability ranking diagram of fourteen outcomes of different interventions.
A: mean pulmonary artery pressure(mPAP) change; B: pulmonary artery systolic pressure(PASP) change; C: pulmonary vascular resistance(PVR) change; D: pulmonary arterial/aortic pressure(PA/AO) change; E: systolic blood pressure(SBP) change; F: heart rate(HR) change; G: blood oxygen saturation(SpO2) change; H: oxygenation index(OI) change; I: partial pressure of arterial oxygen(PaO2) change; J: mechanical ventilation duration; K: intensive care unit stay (ICU) duration; L: Hospital stay duration; M: mortality; N: pulmonary hypertension(PH) crisis.
