# Supplementary material for: Efficacy and safety of endothelin receptor antagonists, phosphodiesterase type 5 Inhibitors, and prostaglandins in pediatric pulmonary arterial hypertension: A network meta-analysis
Source: Front Cardiovasc Med. 2023 Jan 11;9:1055897. doi: 10.3389/fcvm.2022.1055897 (PMC9875131; doi:10.3389/fcvm.2022.1055897)
Supplement: Supplementary file 2 [file Data_Sheet_2.PDF]

Supplementary Table 1. Search expression in each database.

| Database | Search formula |
|---|---|
| Pubmed<br>(385 results) | ((("Pulmonary Arterial Hypertension"[Mesh]) OR ((((Pulmonary Arterial Hypertension[Title/Abstract]) OR (Arterial Hypertension, Pulmonary[Title/Abstract])) OR (Hypertension, Pulmonary Arterial[Title/Abstract])) OR (PAH[Title/Abstract]))) AND ((((("Child"[Mesh]) OR ((Child[Title/Abstract]) OR (Children[Title/Abstract]))) OR (("Child, Preschool"[Mesh]) OR ((((Child, Preschool[Title/Abstract]) OR (Preschool Child[Title/Abstract])) OR (Children, Preschool[Title/Abstract])) OR (Preschool Children[Title/Abstract])))) OR (("Adolescent"[Mesh]) OR (((((((((((((((((Adolescent[Title/Abstract]) OR (Adolescents[Title/Abstract])) OR (Adolescence[Title/Abstract])) OR (Teens[Title/Abstract])) OR (Teen[Title/Abstract])) OR (Teenagers[Title/Abstract])) OR (Teenager[Title/Abstract])) OR (Youth[Title/Abstract])) OR (Youths[Title/Abstract])) OR (Adolescents, Female[Title/Abstract])) OR (Adolescent, Female[Title/Abstract])) OR (Female Adolescent[Title/Abstract])) OR (Female Adolescents[Title/Abstract])) OR (Adolescents, Male[Title/Abstract])) OR (Adolescent, Male[Title/Abstract])) OR (Male Adolescent[Title/Abstract])) OR (Male Adolescents[Title/Abstract])) OR (young people[Title/Abstract])) OR (Young person[Title/Abstract])))) OR (("Infant"[Mesh]) OR ((((Infant[Title/Abstract]) OR (Infants[Title/Abstract])) OR (Baby[Title/Abstract])) OR (Babies[Title/Abstract]))))) AND (((("Prostaglandins"[Mesh]) OR ((((Prostaglandins[Title/Abstract]) OR (Prostanoid[Title/Abstract])) OR (Prostanoids[Title/Abstract])) OR (Prostaglandin[Title/Abstract]))) OR (("Endothelin Receptor Antagonists"[Mesh]) OR (((((((((Endothelin Receptor Antagonists[Title/Abstract]) OR (Antagonists, Endothelin Receptor[Title/Abstract])) OR (Endothelin Antagonists[Title/Abstract])) OR (Antagonists, Endothelin[Title/Abstract])) OR (Endothelin Receptor Antagonist[Title/Abstract])) OR (Antagonist, Endothelin Receptor[Title/Abstract])) OR (Receptor Antagonist, Endothelin[Title/Abstract])) OR (Endothelin Antagonist[Title/Abstract])) OR (Antagonist, Endothelin[Title/Abstract])))) OR (("Phosphodiesterase 5 Inhibitors"[Mesh]) OR ((((((((((((((((Phosphodiesterase 5 Inhibitors[Title/Abstract]) OR (Inhibitors, Phosphodiesterase 5[Title/Abstract])) OR (PDE5 Inhibitor[Title/Abstract])) OR (Inhibitor, PDE5[Title/Abstract])) OR (Phosphodiesterase 5 Inhibitor[Title/Abstract])) OR (5 Inhibitor, Phosphodiesterase[Title/Abstract])) OR (Inhibitor, Phosphodiesterase 5[Title/Abstract])) OR (PDE-5 Inhibitor[Title/Abstract])) OR (Inhibitor, PDE-5[Title/Abstract])) OR (PDE 5 Inhibitor[Title/Abstract])) OR (PDE-5 Inhibitors[Title/Abstract])) OR (Inhibitors, PDE-5[Title/Abstract])) OR (PDE 5 Inhibitors[Title/Abstract])) OR (PDE5 |

| | |
|---|---|
| | Inhibitors[Title/Abstract])) OR (Inhibitors, PDE5[Title/Abstract])) OR (Phosphodiesterase Type 5 Inhibitors[Title/Abstract])) OR (Phosphodiesterase Type 5 Inhibitor[Title/Abstract])))) |
| Embase<br>(2772 results) | ('pulmonary hypertension' OR 'pulmonary arterial hypertension' OR 'arterial hypertension, pulmonary' OR 'hypertension, pulmonary arterial' OR pah) AND ( 'preschool child' OR 'child, preschool' OR 'children, preschool' OR 'preschool children' OR child OR children OR adolescent OR adolescents OR adolescence OR teens OR teen OR teenagers OR teenager OR youth OR youths OR 'adolescents, female' OR 'adolescent, female' OR 'female adolescent' OR 'female adolescents' OR 'adolescents, male' OR 'adolescent, male' OR 'male adolescent' OR 'male adolescents' OR 'young people' OR 'young person') OR infant OR infants OR baby OR babies) AND (prostaglandin OR prostaglandins OR prostanoid OR prostanoids OR 'endothelin receptor antagonist' OR 'endothelin receptor antagonists':ab,ti OR 'antagonists, endothelin receptor' OR 'endothelin antagonists' OR 'antagonists endothelin' OR 'antagonist, endothelin receptor' OR 'receptor antagonist endothelin' OR 'endothelin antagonist' OR 'antagonist endothelin' OR 'phosphodiesterase v inhibitor' OR 'phosphodiesterase 5 inhibitors' OR 'inhibitors, phosphodiesterase 5' OR 'pde5 inhibitor' OR 'inhibitor, pde5' OR 'phosphodiesterase 5 inhibitor' OR '5 inhibitor, phosphodiesterase' OR 'inhibitor, phosphodiesterase 5' OR 'pde-5 inhibitor' OR 'inhibitor, pde-5' OR 'pde 5 inhibitor' OR 'pde-5 inhibitors' OR 'inhibitors, pde-5' OR 'pde 5 inhibitors' OR 'pde5 inhibitors' OR 'inhibitors, pde5' OR 'phosphodiesterase type 5 inhibitors' OR 'phosphodiesterase type 5 inhibitor') |
| Web of Science<br>(270 results) | (Pulmonary Arterial Hypertension (Topic) or Arterial Hypertension, Pulmonary (Topic) or Hypertension, Pulmonary Arterial (Topic) or PAH (Topic)) AND (Child (Topic) or Children (Topic) OR Child, Preschool (Topic) or Preschool Child (Topic) or Children, Preschool (Topic) or Preschool Children (Topic) OR Adolescent (Topic) or Adolescents (Topic) or Adolescence (Topic) or Teens (Topic) or Teen (Topic) or Teenagers (Topic) or Teenager (Topic) or Youth (Topic) or Youths (Topic) or Adolescents, Female (Topic) or Adolescent, Female (Topic) or Female Adolescent (Topic) or Female Adolescents (Topic) or Adolescents, Male (Topic) or Adolescent, Male (Topic) or Male Adolescent (Topic) or Male Adolescents (Topic) or young people (Topic) or Young person (Topic) OR Infant (Topic) or Infants (Topic) or Baby (Topic) or Babies (Topic)) AND (Prostaglandins (Topic) or Prostanoid (Topic) or Prostanoids (Topic) or Prostaglandin (Topic) OR Endothelin Receptor Antagonists (Topic) or Antagonists, Endothelin Receptor (Topic) or Endothelin Antagonists (Topic) or Antagonists, Endothelin (Topic) or Endothelin Receptor Antagonist (Topic) or Antagonist, Endothelin Receptor (Topic) or Receptor Antagonist, Endothelin (Topic) or Endothelin Antagonist (Topic) or |

| | |
|---|---|
| | Antagonist, Endothelin (Topic) OR Phosphodiesterase 5 Inhibitors (Topic) or Inhibitors, Phosphodiesterase 5 (Topic) or PDE5 Inhibitor (Topic) or Inhibitor, PDE5 (Topic) or Phosphodiesterase 5 Inhibitor (Topic) or 5 Inhibitor, Phosphodiesterase (Topic) or Inhibitor, Phosphodiesterase 5 (Topic) or PDE-5 Inhibitor (Topic) or Inhibitor, PDE-5 (Topic) or PDE 5 Inhibitor (Topic) or PDE-5 Inhibitors (Topic) or Inhibitors, PDE-5 (Topic) or PDE 5 Inhibitors (Topic) or PDE5 Inhibitors (Topic) or Inhibitors, PDE5 (Topic) or Phosphodiesterase Type 5 Inhibitors (Topic) or Phosphodiesterase Type 5 Inhibitor (Topic) ) |
| Cochrane Library<br>(110 results) | ((Pulmonary Arterial Hypertension) OR (Arterial Hypertension, Pulmonary) OR (Hypertension, Pulmonary Arterial) OR (PAH)) AND ((Child) OR (Children) OR (Child, Preschool) OR (Preschool Child) OR (Children, Preschool) OR (Preschool Children) OR (Adolescent) OR (Adolescents) OR (Adolescence) OR (Teens) OR (Teen) OR(Teenagers) OR (Teenager) OR (Youth) OR (Youths) OR (Adolescents, Female) OR (Adolescent, Female) OR (Female Adolescent) OR (Female Adolescents) OR (Adolescents, Male) OR (Adolescent, Male) OR (Male Adolescent) OR (Male Adolescents) OR (young people) OR (Young person) OR (Infant) OR (Infants) OR (Baby OR (Babies)) AND ((Prostaglandins) OR (Prostanoid) OR (Prostanoids) OR (Prostaglandin) OR(Endothelin Receptor Antagonists) OR (Antagonists, Endothelin Receptor) OR (Endothelin Antagonists) OR (Antagonists, Endothelin) OR (Endothelin Receptor Antagonist) OR (Antagonist, Endothelin Receptor) OR (Receptor Antagonist, Endothelin) OR (Endothelin Antagonist) OR (Antagonist, Endothelin) OR (Phosphodiesterase 5 Inhibitors) OR (Inhibitors, Phosphodiesterase 5) OR (PDE5 Inhibitor) OR (Inhibitor, PDE5) OR (Phosphodiesterase 5 Inhibitor)OR bitor) OR (5 Inhibitor, Phosphodiesterase) OR (Inhibitor, Phosphodiesterase 5) OR (PDE-5 Inhibitor) OR (Inhibitor, PDE-5) OR (PDE 5 Inhibitor) OR (PDE-5 Inhibitors) OR (Inhibitors, PDE-5) OR (PDE 5 Inhibitors) OR (PDE5 Inhibitors) OR (Inhibitors, PDE5) OR (Phosphodiesterase Type 5 Inhibitors) OR (Phosphodiesterase Type 5 Inhibitor)) |
