# Supplementary material for: Efficacy and safety of endothelin receptor antagonists, phosphodiesterase type 5 Inhibitors, and prostaglandins in pediatric pulmonary arterial hypertension: A network meta-analysis
Source: Front Cardiovasc Med. 2023 Jan 11;9:1055897. doi: 10.3389/fcvm.2022.1055897 (PMC9875131; doi:10.3389/fcvm.2022.1055897)
Supplement: Supplementary file 3 [file Data_Sheet_3.PDF]

Supplementary Table 2. The mPAP change of the included trials.

First author, year	mPAP Change (mean±SD, mmHg)	
	Control	Intervention
Chun-Yan Zhang, 2013	-1.3±15.9	-23.375±13.146
Giordano R, 2015	2.9±1.22	0.5±0.95
Onan IS, 2016	-35.9±10.63	-30.9±8.4
Bhasin S, 2017	-18.5±9.56 (Post-operative Sidenafil)	-23±8.79 (Pre-and post-operative Sidenafil)
Bigdelian H, 2017	-36.8±2.67 (control)	-44.56±3.03 (Post-operative Sidenafil)<br>-45.32±2.58 (Pre- and post-operative Sidenafil)
Shu-Ting Huang, 2020	-16.7±4.35 (Milrinone)	-19.11±3.63 (ProsA)
Patel R, 2020	-22.68±5.22 (Post-operative Sidenafil)	-26.96±5.61 (Pre- and post-operative Sidenafil)
William MB, 2019	-7.9±15.1	-17.4±16.4
Takahashi K, 2003	-5.1±5.67	-5.4±7.5
Farhangdoust S, 2022	-15.88±6.57 (Sidenafil)	-19.15±7.7 (Bosentan)
Zhuming Xu, 2015	18±27.2	-3.3±4.7
Barst RJ, 2011	-0.4±15.9	-4.3±13.9

Supplementary Table 3. The PASP change of the included trials.

First author, year	PASP Change (mean±SD, mmHg)	
	Control	Intervention
Namachivayam P, 2006	5.6±9.53	-1.3±12.62
Peiravian F, 2007	-28.89±14.87	-33.25±16.9
Kahveci H, 2014	-38.2±7.73 (Sidenafil)	-45.8±7.01 (ProsA)
Bhasin S, 2017	-22.499±11.149 (Post-operative Sidenafil)	-26.7±9.97 (Pre- and post-operative Sidenafil)
El-Ghandour M, 2020	-20.5±18.65 (Milrinone)	-16.4±14.64 (Sidenafil)<br>-33.5±20.67 (Milrinone and Sidenafil)
Shu-Ting Huang, 2020	-38.76±8.46 (Milrinone)	-47±9.92 (ProsA)
Zhuming Xu, 2015	19.7±31.2	-3.9±6.2

Supplementary Table 4. The PVR change of the included trials.

First author, year	PVR Change (mean±SD, Wood units)	
	Control	Intervention
Bigdelian H, 2017	-6.53±0.47 (control)	-7.91±0.54 (Post-operative Sidenafil)<br>-8.06±0.48 (Pre- and post-operative Sidenafil)
Takahashi K, 2003	-0.4±1.14	-1.4±1.15
Zhuming Xu, 2015	6.8±10.3	-2.2±3.2

Supplementary Table 5. The PA/AO change of the included trials.

First author, year	PA/AO Change (mean±SD)	
	Control	Intervention
Peiravian F, 2007	-0.46±0.12	-0.61±0.09
Farah P, 2013	-0.52±0.12 (Milrinone)	-0.41±0.13 (Sidenafil)<br>-0.58±0.12 (Milrinone and Sidenafil)
Giordano R, 2015	0.02±0.036	-0.02±0.036
Onan IS, 2016	-0.4±0.13	-0.41±0.176
Bhasin S, 2017	-0.161±0.104 (Post-operative Sidenafil)	-0.53±0.685 (Post-operative Sidenafil)<br>-0.294±0.103 (Pre- and post-operative Sidenafil)
Bigdelian H, 2017	-0.44±0.586 (control)	-0.53±0.685 (Post-operative Sidenafil)<br>-0.56±0.59 (Pre- and post-operative Sidenafil)
Patel R, 2020	-0.272±0.098 (Post-operative Sidenafil)	-0.362±0.054 (Pre- and post-operative Sidenafil)

Supplementary Table 6. The SBP change of the included trials.

First author, year	SBP Change (mean±SD, mmHg)	
	Control	Intervention
Chun-Yan Zhang, 2013	-0.81±5.065	-2.045±15.32
Bhasin S, 2017	-10.4±7.83 (Post-operative Sidenafil)	-6.5±8.982 (Pre- and post-operative Sidenafil)
El-Ghandour M, 2020	1.2±15.1 (Milrinone)	1.65±13.107 (Sidenafil)<br>-0.25±14.51 (Milrinone and Sidenafil)
Shu-Ting Huang, 2020	23.33±4.975 (Milrinone)	19.36±5.79 (ProsA)
William MB, 2019	-1.1±18.84	-3.1±15.15
Zhuming Xu, 2015	11.4±39.5	3.4±22.4

Supplementary Table 7. The HR change of the included trials.

First author, year	HR Change (mean±SD, beats/min)	
	Control	Intervention
Chun-Yan Zhang, 2013	-1.6±10.6	-6.185±9.439
Shu-Ting Huang, 2020	34.39±9.79 (Milrinone)	22.31±10.815 (ProsA)
William MB, 2019	-8.9±21.613	-9.4±24.496
Zhuming Xu, 2015	6.3±31.2	1.9±16.3

Supplementary Table 8. The SpO_2 change of the included trials.

First author, year	SpO_2 Change (mean±SD, %)	
	Control	Intervention
Chun-Yan Zhang, 2013	1.04±1.269	1.225±0.939
Giordano R, 2015	13.5±2.326	15.8±1.61
Bigdelian H, 2017	8.8±0.969 (control)	9.1±1.767 (Post-operative Sidenafil)<br>9.6±0.97 (Pre- and post-operative Sidenafil)
Shu-Ting Huang, 2020	0.76±1 (Milrinone)	0.97±1.013(ProsA)
William MB, 2019	4.6±8.7-2	5.5±11.547

Supplementary Table 9. The OI change of the included trials.

First author, year	OI Change (mean±SD, beats/min)	
	Control	Intervention
Kahveci H, 2014	-34.4±6.585 (Sidenafil)	-33.55±5.88 (ProsA)
Onan IS, 2016	-1.2±0.458	-0.5±0.819
El-Ghandour M, 2020	-6±14.29 (Milrinone)	-6±12.633 (Sidenafil)<br>-11.75±10 (Milrinone and Sidenafil)
Pierce CM, 2021	-9.8±14.73	-11.3±15.38

Supplementary Table 10. The PaO_2 change of the included trials.

First author, year	PaO_2 Change (mean±SD, mmHg)	
	Control	Intervention
Namachivayam P, 2006	10±53.507	8±57.297
Chun-Yan Zhang, 2013	17.15±8.28	29.1±17.309
Onan IS, 2016	2.1±7.119	3.4±9.737
Shu-Ting Huang, 2020	34.28±14.248 (Milrinone)	56.83±16.581 (ProsA)

Supplementary Table 11. The mechanical ventilation duration of the included trials.

First author, year	Duration of ventilation (mean±SD, h)		
	Control	Intervention	p value
Namachivayam P, 2006	98±130.7	28.2±28.81(Sidenafil)*	p＝0.018
Peiravian F, 2007	22.6±9.5	13.75±12.12(Sidenafil)*	p＝0.013
Vargas-Origel A, 2010	72±363.7	120±260.7	-
Uslu S, 2011	144±177.8	96±142.2(Sidenafil) *	p＝0.001
Mohamed WA, 2012	274±14.4	103.2±21.6(Bosentanl)*	p＜0.001
Kahveci H, 2014	247.2±102.72 (Sidenafil)	149.52±81.36 (ProsA) *	p＝0.000
Giordano R, 2015	23.3±6	15.5±4(Sidenafil) *	p＝0.0004
Sharma VK, 2015	22.1±10.6	7±7.24(Sidenafil)*	-
Al Omar S, 2016	161.28±103.92	245.52±192	-
Onan IS, 2016	105.6±148.8	98.4±136.8	-
Steinhorn RH, 2016	206.4±85.4	259.2±178.72	-
Bhasin S, 2017	28.067±15.422 (Post-operative Sidenafil)	23.233±13.868 (Pre- and post-operative Sidenafil)	-
Bigdelian H, 2017	12.3±1 (control)	9.07±1.3 (Post-operative Sidenafil)*<br>8.4±0.9 (Pre- and post-operative Sidenafil) *	p＝0.001<br>p＝0.001
Shu-Ting Huang, 2020	59.76±19.68 (Milrinone)	49.92±20.16(ProsA) *	p＝0.03
Patel R, 2020	30.53±13.05 (Post-operative Sidenafil)	22.79±17.13 (Pre- and post-operative Sidenafil) *	p＝0.04
Thandaveshwara D, 2021	240±32	192±97.78	-

Supplementary Table 12. The duration of ICU stay of the included trials.

First author, year	Duration of ICU stay (mean±SD, h)		
	Control	Intervention	p value
Namachivayam P, 2006	189±173.9	47.8±69.78(Sidenafil) *	p＜0.004
Peiravian F, 2007	86.28±93.94	72.65±29.97	-
Farah P, 2013	68±25 (Milrinone)	108±65 (Sidenafil)*<br>120±78 (Milrinone and Sidenafil) *	p＜0.02<br>p＜0.01
Giordano R, 2015	109±16	86±10(Sidenafil) *	p＝0.0001
Sharma VK, 2015	64.4±15.9	42.3±8.8(Sidenafil)*	-
Onan IS, 2016	199.2±172.8	175.2±153.6	-
Bhasin S, 2017	98.4±18.34 (Post-operative Sidenafil)	78.467±19.501 (Pre- and post-operative Sidenafil) *	p＝0.001
Bigdelian H, 2017	75.3±3 (control)	70.71±2 (Post-operative Sidenafil)*<br>70.09±4.7 (Pre- and post-operative Sidenafil) *	p＝0.001<br>p＝0.001
Shu-Ting Huang, 2020	114.48±21.26 (Milrinone)	76.56±22.08 (ProsA)*	p＝0.007

Patel R, 2020	80±42.59 (Post operative Sidenafil)	66±30 (Pre- and post-operative Sidenafil)	-

Supplementary Table 13. The duration of hospital stay of the included trials.

First author, year	Duration of hospital stay (mean±SD, day)		
	Control	Intervention	p value
Peiravian F, 2007	7.8±3.78	7.2±2.14	-
Farah P, 2013	5.5±1.7 (Milrinone)	8.5±6.4 (Sidenafil)<br>7.9±4.7 (Milrinone and Sidenafil)	-
Al Omar S, 2016	17.81±11.5	23.2±15.2	-
Onan IS, 2016	15±7.5	15.3±9.3	-
Shu-Ting Huang, 2020	10.24±1.78 (Milrinone)	8.81±2.16 (ProsA)*	p＝0.01
Patel R, 2020	7.53±1.92 (Post-operative Sidenafil)	6.13±1.4(Pre- and post-operative Sidenafil) *	p＝0.05
William MB, 2019	6.9±4.8	8.7±7.2	-
Thandaveshwara D, 2021	13±3.074	13±13.33	-
Fatima N, 2018	10.12±5.2 (Sidenafil)	7.56±3.77 (Sidenafil and Bosentan) *	p＜0.0001

Supplementary Table 14. The mortality of the included trials.

First author, year	Mortality (mean±SD, responders)		
	Control	Intervention	p value
Vargas-Origel A, 2010	8	2(Sidenafil)*	p＜0.004
Uslu S, 2011	2	1	-
Mohamed WA, 2012	3	1	-
Kahveci H, 2014	4 (Sidenafil)	3 (ProsA)	-
Giordano R, 2015	1	0	-
El-Ghandour M, 2020	6 (Milrinone)	5 (Sidenafil)<br>3 (Milrinone and Sidenafil)	-
William MB, 2019	21	13	-
Pierce CM, 2021	1	2	-
Thandaveshwara D, 2021	5	2	-
Bagheri MM, 2021	4 (Milrinone)	3 (Milrinone and Sidenafil)	-

Supplementary Table 15. The PH crisis of the included trials.

First author, year	PH crisis (mean±SD, responders)		
	Control	Intervention	p value
Peiravian F, 2007	4	0(Sidenafil) *	p＝0.02
Farah P, 2013	0 (Milrinone)	6 (Sidenafil)*<br>3 (Milrinone and Sidenafil)	p＝0.01<br>-

Onan IS, 2016	2	4	-
Bhasin S, 2017	1 (Post-operative Sidenafil)	2 (Pre- and post-operative Sidenafil)	-
Bigdelian H, 2017	3(control)	0 (Post operative Sidenafil)<br>0 (Pre- and post-operative Sidenafil)	-

mPAP: mean pulmonary artery pressure; PASP: pulmonary artery systolic pressure; PVR: pulmonary vascular resistance; PA/AO: pulmonary arterial/aortic pressure; SBP: systolic blood pressure; HR: heart rate; SpO_2 : blood oxygen saturation; OI: oxygenation index; PaO_2 : partial pressure of arterial oxygen; ICU: intensive care unit; PH: pulmonary hypertension.

* represents a statistical difference between the intervention group and the control group in supplementary Table 11-15.
