# Supplementary material for: Efficacy and safety of endothelin receptor antagonists, phosphodiesterase type 5 Inhibitors, and prostaglandins in pediatric pulmonary arterial hypertension: A network meta-analysis
Source: Front Cardiovasc Med. 2023 Jan 11;9:1055897. doi: 10.3389/fcvm.2022.1055897 (PMC9875131; doi:10.3389/fcvm.2022.1055897)
Supplement: Supplementary file 4 [file Data_Sheet_4.PDF]

Supplementary Table 16. The Node-Splitting Method Specific Results From Network Meta-Analysis

Treatment	PASP	PA/AO	Duration of ventilation	ICU stay duration
ProsA vs Control	0.498	-	0.107	0.308
Sildenafil vs Control	0.498	-	0.137	0.310
ProsA vs Milrinone	0.779	-	-	0.300
Sildenafil vs Milrinone	0.763	-	-	0.302
Sildenafil vs ProsA	0.514	-	0.113	-
Post-operative Sildenafil vs Control	-	0.639	0.863	0.959

PASP: pulmonary artery systolic pressure; PA/AO: pulmonary arterial/aortic pressure; ICU: intensive care unit.
