# Supplementary material for: Efficacy and safety of endothelin receptor antagonists, phosphodiesterase type 5 Inhibitors, and prostaglandins in pediatric pulmonary arterial hypertension: A network meta-analysis
Source: Front Cardiovasc Med. 2023 Jan 11;9:1055897. doi: 10.3389/fcvm.2022.1055897 (PMC9875131; doi:10.3389/fcvm.2022.1055897)
Supplement: Supplementary file 5 [file Data_Sheet_5.PDF]

Supplementary Table 17. Weighted Mean Difference(95%CI) For mPAP From Network Meta-Analysis

Bosentan					
-9.73 (-30.24, 10.44)	Control				
-5.10 (-33.12, 24.56)	4.63 (-15.08, 26.17)	Milrinone			
-5.74 (-26.76, 15.04)	4.00 (-8.20, 16.11)	-0.62 (-25.51, 22.35)	Post-operative Sildenafil		
-2.69 (-24.72, 20.82)	7.10 (-2.41, 18.03)	2.42 (-15.48, 20.23)	3.08 (-11.95, 19.80)	ProsA	
-3.29 (-21.67, 14.99)	6.46 (-2.34, 15.42)	1.84 (-21.33, 23.30)	2.46 (-7.52, 12.61)	-0.62 (-14.94, 12.17)	Sildenafil

Supplementary Table 18. Weighted Mean Difference(95%CI) For PASP From Network Meta-Analysis

Control			
1.37 (-10.28, 13.06)	Post-operative Sildenafil		
3.20 (-4.52, 13.20)	1.65 (-11.57, 17.55)	ProsA	
1.53 (-10.03, 13.25)	0.15 (-11.36, 11.72)	-1.48 (-17.43, 11.87)	Sildenafil

Supplementary Table 19. Weighted Mean Difference(95%CI) For PVR From Network Meta-Analysis

Control			
1.37 (-10.13, 12.95)	Post-operative Sildenafil		
3.15 (-4.44, 13.13)	1.60 (-11.46, 17.48)	ProsA	
1.52 (-10.03, 13.08)	0.15 (-11.41, 11.67)	-1.42 (-17.40, 11.60)	Sildenafil

Supplementary Table 20. Weighted Mean Difference(95%CI) For PA/AO From Network Meta-Analysis

Control					
0.20(-0.02, 0.44)	Milrinone				
0.26 (0.04, 0.50)	0.06 (-0.13, 0.25)	Milrinone and Sildenafil			
-0.02 (-0.16, 0.17)	-0.21 (-0.44, 0.02)	-0.27(-0.50, -0.04)	Post-operative Sildenafil		
0.01 (-0.20, 0.22)	-0.19 (-0.51, 0.11)	-0.25 (-0.57, 0.05)	0.03 (-0.25, 0.27)	ProsA	
0.09 (-0.02, 0.22)	-0.11 (-0.30, 0.09)	-0.17 (-0.36, 0.03)	0.11 (-0.02, 0.22)	0.08 (-0.15, 0.33)	Sildenafil

Supplementary Table 21. Weighted Mean Difference(95%CI) For SBP From Network Meta-Analysis

Control					
-0.38 (-10.21, 10.27)	Milrinone				
2.13(-10.97, 15.54)	2.46 (-9.44, 14.04)	Milrinone and Sildenafil			
4.91 (-8.14, 18.07)	5.31(-8.88, 18.59)	2.80 (-12.24, 17.61)	Post-operative Sildenafil		
3.06 (-5.69, 12.02)	3.49 (-5.08, 11.33)	0.94 (-12.30, 14.14)	-1.86 (-15.88, 12.48)	ProsA	
1.03 (-7.84, 9.77)	1.38 (-8.79, 10.78)	-1.10 (-12.73, 10.15)	-3.91 (-13.63, 5.71)	-2.06 (-12.58, 8.15)	Sildenafil

Supplementary Table 22. Weighted Mean Difference(95%CI) For HR From Network Meta-Analysis

Control			
-7.51 (-27.18, 12.23)	Milrinone		
4.54 (-8.39, 17.47)	12.04 (-2.80, 26.89)	ProsA	
0.45 (-15.16, 16.10)	7.98 (-17.20, 33.13)	-4.078 (-24.40, 16.19)	Sildenafil

Supplementary Table 23. Weighted Mean Difference(95%CI) For SpO_2 From Network Meta-Analysis

Control				
0.02 (-3.77, 3.78)	Milrinone			
-0.53 (-3.11, 1.95)	-0.54 (-5.16, 3.99)	Post-operative Sildenafil		
-0.19 (-2.89, 2.50)	-0.21 (-2.85, 2.46)	0.34 (-3.33, 4.11)	ProsA	
-1.26 (-3.11, 0.38)	-1.26 (-5.54, 2.80)	-0.73 (-3.32, 1.74)	-1.06 (-4.39, 2.04)	Sildenafil

Supplementary Table 24. Weighted Mean Difference(95%CI) For OI From Network Meta-Analysis

Control				
0.62 (-11.29, 12.98)	Milrinone			
6.38 (-4.68, 17.95)	5.79 (-3.92, 15.39)	Milrinone and Sildenafil		
-0.61 (-6.00, 5.24)	-1.15 (-13.05, 10.60)	-6.92 (-18.00, 3.92)	ProsA	
0.66 (-5.78, 7.55)	0.04 (-10.16, 10.17)	-5.72 (-15.00, 3.45)	1.21 (-4.75, 7.32)	Sildenafil

Supplementary Table 25. Weighted Mean Difference(95%CI) For PaO_2 From Network Meta-Analysis

Control			
16.43 (-17.40, 49.43)	Milrinone		
-6.15 (-25.93, 12.78)	-22.57 (-49.75, 4.69)	ProsA	
1.81 (-45.08, 49.04)	-14.50 (-70.85, 42.58)	8.07 (-41.91, 59.02)	Sildenafil

Supplementary Table 26. Weighted Mean Difference(95%CI) For duration of mechanical ventilation From Network Meta-Analysis

Bosentan					
-166.15 (-182.00, -118.81)	Control				
-87.73 (-143.60, -20.39)	77.24 (20.26, 132.02)	Milrinone			
-161.46 (-179.82, -110.07)	4.73 (-8.45, 19.60)	-72.40 (-127.72, -14.27)	Post-operative Sildenafil		
-77.61 (-130.41, -15.51)	87.09 (35.36, 137.52)	9.90 (-12.00, 32.27)	82.26 (29.10, 133.61)	ProsA	
-157.04 (-173.88, -106.11)	9.04 (1.32, 20.42)	-67.89 (-122.05, -10.73)	4.29 (-6.68, 17.41)	-77.68 (-127.69, -25.85)	Sildenafil

Supplementary Table 27. Weighted Mean Difference(95%CI) For duration of ICU stay From Network Meta-Analysis

control					
51.55(3.39, 98.73)	Milrinone				
1.47 (-59.36, 62.69)	-50.14 (-101.32, 3.31)	Milrinone and Sildenafil			
6.31 (-16.60, 32.92)	-45.10 (-93.21, 5.90)	5.05 (-56.80, 67.42)	Post-operative Sildenafil		
86.32 (27.25, 138.31)	35.12 (-4.97, 65.89)	84.93 (18.51, 142.73)	79.85 (16.54, 133.16)	ProsA	
18.63 (3.22, 39.70)	-32.60 (-76.35, 14.95)	17.59 (-40.65, 77.43)	12.28 (-7.38, 34.49)	-67.35 (-117.17, -6.91)	Sildenafil

Supplementary Table 28. Weighted Mean Difference(95%CI) For duration of hospital stay From Network Meta-Analysis

Control						
0.98 (-5.46, 6.38)	Milrinone					
-1.17 (-8.52, 5.46)	-2.16 (-7.87, 3.86)	Milrinone and Sildenafil				
-2.44 (-9.83, 3.85)	-3.50 (-11.26, 4.51)	-1.34 (-9.89, 7.11)	Post-operative Sildenafil			
1.94 (-4.88, 7.30)	1.05 (-4.33, 5.68)	3.17 (-4.57, 9.91)	4.51 (-4.29, 12.45)	ProsA		
-1.08 (-5.27, 2.11)	-2.09 (-7.41, 3.42)	0.05 (-6.31, 6.33)	1.40(-4.35, 7.14)	-3.08 (-8.80, 3.40)	Sildenafil	
1.48(-6.00, 7.84)	0.45 (-7.43, 8.49)	2.61 (-6.03, 11.09)	3.94(-4.25, 12.15)	-0.55 (-8.62, 8.27)	2.54 (-3.30, 8.35)	Sildenafil and Bosentan

Supplementary Table 29. Weighted Mean Difference(95%CI) For mortolity From Network Meta-Analysis

Bosentan					
-2.13 (-6.09, 1.15)	Control				
-1.59 (-6.30, 2.89)	0.57 (-2.13, 3.53)	Milrinone			
-0.92 (-5.69, 3.58)	1.23 (-1.53, 4.24)	0.65 (-1.30, 2.63)	Milrinone and Sildenafil		
-1.43 (-6.30, 3.21)	0.71 (-2.25, 3.98)	0.14 (-3.65, 3.91)	-0.52 (-4.35, 3.32)	ProsA	
-1.41 (-5.51, 2.20)	0.71 (-0.43, 2.11)	0.14 (-2.39, 2.66)	-0.52 (-3.13, 2.06)	-5.40 (-2.87, 2.80)	Sildenafil

Supplementary Table 30. Weighted Mean Difference(95%CI) For PH crisis From Network Meta-Analysis

Control					
53.08(13.91, 110.51)	Milrinone				
27.25 (4.56, 81.92)	-19.98(-62.66, -1.94)	Milrinone and Sildenafil			
27.12 (4.52, 81.77)	-20.21 (-62.85, -1.26)	-0.14 (-6.12, 6.19)	Post-operative Sildenafil		
-0.73(-4.98, 3.43)	-53.80 (-111.41, -14.31)	-28.02 (-82.58, -4.73)	-27.96 (-82.50, -4.68)	ProsA	
26.05 (3.98, 80.68)	-21.07(-63.73, -3.10)	-1.04 (-5.11, 3.02)	-0.90 (-5.85, 3.54)	26.93 (4.15, 81.39)	Sildenafil

mPAP: mean pulmonary artery pressure; PASP: pulmonary artery systolic pressure; PVR: pulmonary vascular resistance; PA/AO: pulmonary arterial/aortic pressure; SBP: systolic blood pressure; HR: heart rate; SpO_2 : blood oxygen saturation; OI: oxygenation index; PaO_2 : partial pressure of arterial oxygen; ICU: intensive care unit; PH: pulmonary hypertension.
